# Association between time to advanced airway management and survival during pediatric out-of-hospital cardiac arrest

**DOI:** 10.1016/j.resplu.2022.100260

**Published:** 2022-06-24

**Authors:** Naoko Ohashi-Fukuda, Tatsuma Fukuda, Kent Doi

**Affiliations:** a Department of Emergency and Critical Care Medicine, Graduate School of Medicine, The University of Tokyo, Tokyo, Japan; b Department of Emergency and Critical Care Medicine, Toranomon Hospital, Tokyo, Japan; c Department of Emergency and Critical Care Medicine, Graduate School of Medicine, University of the Ryukyus, Okinawa, Japan

**Keywords:** Out-of-hospital cardiac arrest, Cardiopulmonary resuscitation, Advanced airway management, Children, Pediatrics

## Abstract

**Background:**

Respiratory care, including advanced airway management (AAM), is an important part of pediatric resuscitation. This study aimed to determine whether time to AAM is associated with outcomes after out-of-hospital cardiac arrest (OHCA) in children.

**Methods:**

This was a nationwide population-based observational study using the Japanese government-led registry of OHCA patients. Children (aged 1–17 years) who experienced OHCA and received AAM by emergency medical service (EMS) personnel in the prehospital setting from 2014 to 2019 were included. Multivariable logistic regression models were used to assess the associations between time to AAM (defined as time in minutes from emergency call to the first successful AAM) and outcomes after OHCA. The primary outcome was one-month overall survival. The secondary outcomes were prehospital return of spontaneous circulation (ROSC) and one-month neurologically favorable survival.

**Results:**

A total of 761 patients (mean [SD] age, 12.7 [4.8] years) were included. The mean time to AAM was 18.9 min (SD, 7.9). Overall, 77 (10.1%) patients survived one month after OHCA. After adjusting for potential confounders, longer time to AAM was significantly associated with a decreased chance of one-month survival (multivariable adjusted OR per minute delay, 0.93 [95% CI, 0.89–0.97]; P = 0.001). Similar association was observed for prehospital ROSC (adjusted OR, 0.94 [95% CI, 0.90–0.99]; P = 0.01) and neurologically favorable survival (adjusted OR, 0.83 [95% CI, 0.72–0.95]; P = 0.006). This association between time to AAM and survival was consistent across a variety of sensitivity and subgroup analyses.

**Conclusions:**

Among pediatric OHCA patients, delayed AAM was associated with a decreased chance of survival, although the influence of resuscitation time bias might remain.

## Introduction

The pediatric out-of-hospital cardiac arrest (OHCA) population accounts for nearly 1% of 120,000 OHCA cases every year in Japan.1., 2., 3., 4. Approximately 10–20% of OHCA patients survive one month after OHCA.3., 4., 5., 6., 7. Despite gradual improvements, survival rates after pediatric OHCA remain poor.3., 5.

Advanced airway management (AAM) is a method used in the pediatric ALS algorithm, which consists of endotracheal tube (ETT) or supraglottic airway [SGA] (e.g., laryngeal mask airway [LMA] or laryngeal tube [LT]) insertion. This may improve ventilation, reduce the risk of aspiration, and enable uninterrupted compression delivery. However, in some cases, the procedure may be difficult to complete and may interrupt the delivery of compressions or result in malpositioning of the device. In addition, AAM in children requires specialized equipment and skilled providers, which may be difficult for professionals who do not routinely intubate children.^7^

Prehospital AAM may have an important role in pediatric OHCA because of the respiratory nature of the majority of events.8., 9., 10., 11. However, thus far, no randomized controlled trials (RCTs) have studied the effect of prehospital AAM in pediatric OHCA. A pseudo-RCT, including pediatric patients with acute respiratory problems (including OHCA), indicated that prehospital endotracheal intubation (ETI) might not improve survival or neurological outcomes.^12^ Our previous observational study indicated that prehospital AAM was not associated with an increased chance of one-month survival compared with bag-valve-mask (BVM) ventilation.^4^ In adult OHCA, some studies also suggest that there may not be difference between basic and advanced airway management,13., 14., 15. while other studies have indicated that AAM might be associated with poor neurological outcomes or decreased survival.16., 17., 18. The effectiveness of AAM may vary depending on the timing when AAM is performed. Our previous study examining whether time to AAM is associated with outcomes after OHCA in adults showed that delay in AAM was associated with a decreased chance of one-month survival.^19^

This study aimed to examine the association between the timing of AAM and outcomes after OHCA in children using a large data set from Japan.

## Methods

### Study design, setting and data source

This was a retrospective cohort study using data from the All-Japan Utstein Registry, a government-led nationwide population-based registry of OHCA patients managed by the Fire and Disaster Management Agency (FDMA). In Japan, emergency medical service (EMS) personnel transport all OHCA patients, including those with do-not-resuscitate orders, to an emergency hospital and collect their data using a Utstein-style registry template.16., 20., 21., 22., 23. These data are integrated into the All-Japan Utstein Registry system on the FDMA database server. Rigorous confirmation of the FDMA and the logical internal checks using standardized software ensured the integrity, accuracy, and completeness of the data.

### Emergency medical service in Japan and study population

EMS personnel in Japan perform CPR according to Japanese CPR guidelines, which basically conform to the American Heart Association CPR guidelines, although EMS personnel have different levels of authority depending on their completed training programs.^5^ Most ambulances have three crews, including at least one emergency lifesaving technician (ELST). These highly trained EMS personnel have been certified to provide some part of the advanced life support (ALS) procedures (e.g., insertion of an airway adjunct or an SGA device since 1991). Since 2004, specially trained ELST who has completed additional training sessions and has experienced a certain number of successful intubations are permitted to insert an ETT. As of 2019, almost all ambulances were accompanied by an ELST who could perform SGA insertion, and approximately half of ELSTs were specially trained ELSTs who could perform ETI.^5^ EMS personnel perform prehospital airway management according to a protocol fixed by each municipality (i.e., detailed protocols could vary among municipalities). In principle, bag-mask ventilation is initially delivered, regardless of whether AAM is eventually provided. The decision to subsequently implement AAM depends on the instructions of the medical directors. The choice of airway device used (ETT or SGA) depends on the patient condition and/or the skill of the EMS personnel. Multiple devices are not usually used. For EMS personnel, an attempt to insert an advanced airway device many times or to implement AAM after the return of spontaneous circulation (ROSC) is generally not allowed. In some municipalities, ETI is restricted to patients aged >8 years. In addition to AAM, trained EMS personnel can perform intravenous catheter insertion, fluid infusion, and epinephrine administration as ALS procedures.

**Fig. 1:**
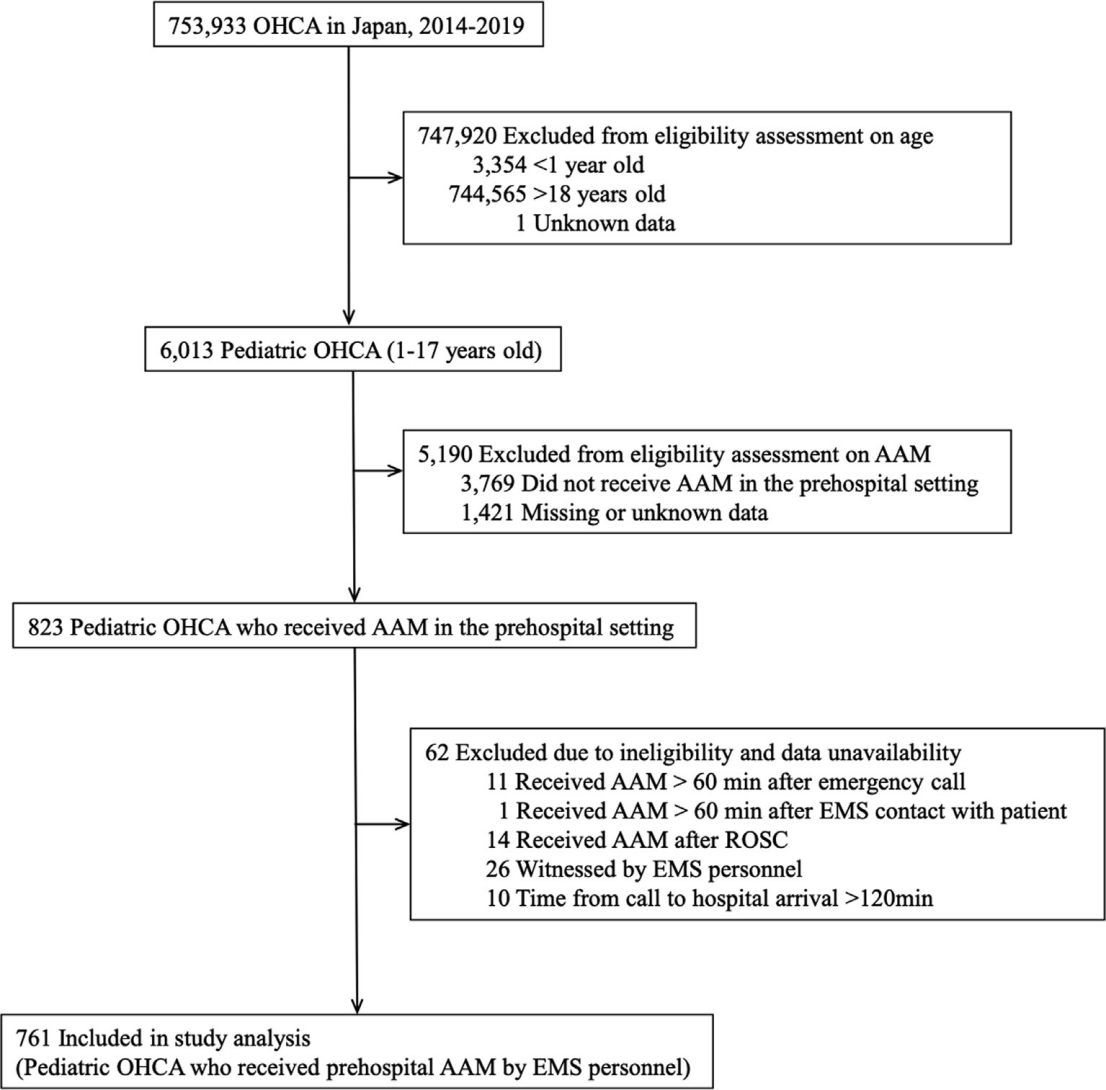
**Patient selection.** Abbreviations: AAM, Advanced airway management; EMS, Emergency medical service; OHCA, Out-of-hospital cardiac arrest; ROSC, Return of spontaneous circulation.

This study included OHCA patients submitted to the All-Japan Utstein Registry between January 1, 2014 and December 31, 2019. We included pediatric OHCA patients aged <18 years, but excluded neonates and infants (<1 year old) due to potential differences in physiological characteristics and etiology of cardiac arrest, who received AAM by EMS personnel in the prehospital setting. Patients in whom advanced airway placement was attempted and was unsuccessful were not included in this study because unsuccessful attempts were not recorded in this registry. We excluded patients who did not receive timely prehospital treatment (time from call to AAM >60 min or time from EMS contact to AAM >60 min), patients who received AAM after ROSC, and patients who did not receive timely in-hospital treatment (time from call to hospital arrival >120 min). Patients with missing, incomplete, or inconsistent data, which accounted for approximately 20% of all pediatric OHCA patients (Fig. 1), were also excluded. This study was conducted in accordance with the amended Declaration of Helsinki. The institutional review boards of the University of Tokyo (2021134NI), Toranomon Hospital (2226), and University of the Ryukyus (1482-R1) approved this study and waived the requirement for documentation of informed consent because of the anonymous nature of the data.

### Data collection

Data on patient characteristics (i.e., age and gender), bystander characteristics (i.e., witness, lay rescuer, bystander CPR, public-access defibrillation, and dispatcher's instruction for CPR), cardiac arrest characteristics (i.e., initial rhythm and etiology of arrest), event characteristics (i.e., year of arrest, time and place of arrest; for seasons and regions, the classification defined by the Japan Meteorological Agency was used), and prehospital ALS characteristics (i.e., AAM, type of advanced airway device, and physician involvement in prehospital ALS) were collected. Data on a series of EMS activity times (i.e., emergency call, contact with patient, AAM, and hospital arrival) were recorded by each EMS squad. Only successful AAM attempts were recorded in the database, whereas failed AAM attempts were not recorded. Time to AAM was defined as the time interval from emergency call to the first successful AAM. Response time represents the time interval between emergency call and contact with patient. Transport time represents the time interval between contact with patient and hospital arrival. Because time intervals were calculated based on time variables recorded in whole minutes, the calculated time interval of 0 min indicates that two events occurred within the same whole minute, and the time interval of 1 min indicates that one event occurred within the next minute after the previous event. Whether ROSC was achieved before arriving at the hospital and the time of ROSC were also recorded. A one-month follow-up survey was conducted by each fire department to collect data on survival and neurological status, based on an inquiry for the receiving hospitals. In addition, the etiology of cardiac arrest was reconfirmed. If the patient was transferred or discharged from the hospital within one month, further investigations were conducted by the fire department in cooperation with hospital personnel.

### Outcomes

The primary outcome was one-month overall survival after OHCA, and the secondary outcomes were prehospital ROSC and one-month neurologically favorable survival, defined as a Glasgow–Pittsburg cerebral performance category (CPC) score of 1 or 2.^23^

### Statistical analysis

Patients were categorized into 1 of 5 pre-specified groups based on the timing of the first successful AAM: ≤6 min, 7–12 min, 13–18 min, 19–24 min, and >24 min. Descriptive statistics were used to characterize all patients and groups according to time to AAM. Categorical variables were presented as counts with proportions. Continuous variables were presented as means with standard deviations (SDs).

To examine the unadjusted association between time to AAM and outcomes after OHCA, the Cochrane-Armitage trend test was used when time to AAM was treated as a categorical variable, and a univariable logistic regression model was used when time to AAM was treated as a linear and continuous variable. To assess the independent association between time to AAM and outcomes after OHCA, multivariable logistic regression models were used. For multivariable logistic regression models, we treated time to AAM as both a categorical and continuous variable. To avoid overfitting, the number of variables included in the model was determined with an event per variable (EPV) of 10 as a guide.^24^ The included variables were selected based on background knowledge.^24^, 25., 26. The following variables were included in the model (Model 1): gender, witness, bystander CPR, public-access defibrillation, dispatcher's instruction for CPR, initial rhythm, etiology of arrest, and type of advanced airway device (LMA, LT, or ETT). We conducted preplanned sensitivity analyses in which different sets of variables were included in the model (Models 2 and 3). In Model 2, included variables were selected based on univariable screening with the EPV of 10 as a guide.^27^ In Model 3, all variables that were available from the registry and could influence the outcomes after OHCA were included, regardless of the EPV.28., 29. We also conducted a preplanned subgroup analysis according to age (<8 or ≥8 years), as resuscitation procedures may sometimes differ between <8 years and ≥8 years.^30^ In addition, to further analyze the association between time to AAM and survival after OHCA in detail, we performed an ancillary analysis in which time to AAM, treated as a linear and continuous variable, was divided into response time and procedure time (i.e., time from contact with patient to AAM). Odds ratios (ORs) with 95% confidence intervals (CIs) were reported.

JMP Pro 16.0.0 software (SAS Institute Inc., Cary, NC, USA) was used to conduct statistical analyses. A two-sided P value of 0.05 was considered statistically significant for all hypothesis tests.

## Results

There were 6,013 pediatric patients with OHCA during the study period, and we identified 761 eligible patients who received AAM by EMS personnel (Fig. 1). The baseline characteristics are summarized in Table 1. The mean age was 12.7 (SD, 4.8) years. The proportion of boys was higher than that of girls (512 [67.3%] vs. 249 [32.7%], respectively). Most patients (584 [76.7%]) had a non-cardiac cause. LT was the most frequently used advanced airway device (603 [79.2%]). The mean time to AAM was 18.9 (SD, 7.9) min.

**Table 1:** Baseline characteristics according to 6-minute intervals of time to advanced airway management in the full cohort.

	All patients	Time to AAM				
		≤6 min	7–12 min	13–18 min	19–24 min	>24 min
	n = 761	n = 4	n = 138	n = 296	n = 173	n = 150
Baseline characteristics						
Age, y						
- Mean (SD)	12.7 (4.8)	12.0 (6.9)	11.7 (5.2)	12.1 (5.0)	13.7 (4.0)	13.3 (4.6)
Age group						
1) <8y - No. (%)	135 (17.7)	1 (25.0)	33 (23.9)	62 (21.0)	18 (10.4)	21 (14.0)
2) ≥ 8y - No. (%)	626 (82.3)	3 (75.0)	105 (76.1)	234 (79.1)	155 (89.6)	129 (86.0)
Gender						
1) Male - No. (%)	512 (67.3)	2 (50.0)	94(68.1)	196(66.2)	127 (73.4)	93 (62.0)
2) Female - No. (%)	249 (32.7)	2 (50.0)	44 (31.9)	100 (33.8)	46 (26.6)	57 (38.0)
Witness						
1) No witness - No. (%)	521 (68.5)	2 (50.0)	110 (79.7)	218 (73.7)	99 (57.2)	92 (61.3)
2) By family member - No. (%)	107 (14.1)	1 (25.0)	13 (9.4)	38 (12.8)	34 (19.7)	21 (14.0)
3) By non-family member - No. (%)	133(17.5)	1 (25.0)	15 (10.9)	40 (13.5)	40 (23.1)	37 (24.7)
Bystander CPR						
1) Yes - No. (%)	492 (64.7)	1 (25.0)	105 (76.1)	207 (69.9)	111 (64.2)	68 (45.3)
2) No - No. (%)	269 (35.3)	3 (75.0)	33 (23.9)	89 (30.1)	62 (35.8)	82 (54.7)
Public-access defibrillation						
1) Yes - No. (%)	12 (1.6)	0 (0.0)	2 (1.5)	2 (0.7)	6 (3.5)	2 (1.3)
2) No - No. (%)	749 (98.4)	4 (100.0)	136 (98.6)	294 (99.3)	167 (96.5)	148 (98.7)
Dispatcher's instruction for CPR						
1) Yes - No. (%)	457 (60.1)	2 (50.0)	98 (71.0)	198 (66.9)	102 (59.0)	57 (38.0)
2) No - No. (%)	304 (40.0)	2 (50.0)	40 (29.0)	98 (33.1)	71 (41.0)	93 (62.0)
Prehospital ALS						
1) ALS by EMS personnel - No. (%)	648 (85.2)	4 (100.0)	117 (84.8)	255 (86.2)	154 (89.0)	118 (78.7)
2) ALS by physician - No. (%)	113 (14.9)	0 (0.0)	21 (15.2)	41 (13.9)	19 (11.0)	32 (21.3)
Initial rhythm						
Shockable rhythm	33 (4.3)	0 (0.0)	6 (4.4)	12 (4.1)	11 (6.4)	4 (2.7)
1) VF - No. (%)	32 (4.2)	0 (0.0)	6 (4.4)	11 (3.7)	11 (6.4)	4 (2.7)
2) VT - No. (%)	1 (0.1)	0 (0.0)	0 (0.0)	1 (0.3)	0 (0.0)	0 (0.0)
Non-shockable rhythm	724 (95.1)	4 (100.0)	132 (95.7)	282 (95.3)	161 (93.1)	145 (96.7)
3) PEA - No. (%)	140 (18.4)	0 (0.0)	26 (18.8)	55 (18.6)	38 (22.0)	21 (14.0)
4) Asystole - No. (%)	584 (76.7)	4 (100.0)	106 (76.8)	227 (76.7)	123 (71.1)	124 (82.7)
Others	4 (0.5)	0(0.0)	0 (0.0)	2 (0.7)	1 (0.6)	1 (0.7)
5) Others (e.g., Bradycardia) - No. (%)	4 (0.5)	0(0.0)	0 (0.0)	2 (0.7)	1 (0.6)	1 (0.7)
Etiology of arrest						
1) Cardiac cause - No. (%)	177 (23.3)	1 (25.0)	37 (26.8)	75 (25.3)	37 (21.4)	27 (18.0)
2) External cause (e.g., asphyxia, drowning, or anaphylaxis) - No. (%)	425(55.9)	1 (25.0)	66 (47.8)	151 (51.0)	98 (56.7)	109 (72.7)
3) Non-cardiac non-external cause (e.g., stroke, respiratory disease, or malignancy) - No. (%)	159(20.9)	2 (50.0)	35 (25.4)	70 (23.7)	38 (22.0)	14 (9.3)
Year of arrest						
1) 2014 - No. (%)	148 (19.5)	1 (25.0)	31 (22.5)	56 (18.9)	32 (18.5)	28 (18.7)
2) 2015 - No. (%)	142 (18.7)	2 (50.0)	23 (16.7)	50 (16.9)	38 (22.0)	29 (19.3)
3) 2016 - No. (%)	122 (16.0)	1 (25.0)	18 (13.0)	45 (15.2)	29 (16.8)	29 (19.3)
4) 2017 - No. (%)	116 (15.2)	0 (0.0)	20 (14.5)	53 (17.9)	21 (12.1)	22 (14.7)
5) 2018 - No. (%)	107 (14.1)	0 (0.0)	20 (14.5)	48 (16.2)	26 (15.0)	13 (8.7)
6) 2019 - No. (%)	126 (16.6)	0 (0.0)	26 (18.8)	44 (14.9)	27 (15.6)	29 (19.3)
Season of arrest						
1) Spring (March, April, May) - No. (%)	194 (25.5)	0 (0.0)	43 (31.2)	75 (25.3)	48 (27.8)	28 (18.7)
2) Summer (June, July, August) - No. (%)	185 (24.3)	3 (75.0)	34 (24.6)	66 (22.3)	39 (22.5)	43 (28.7)
3) Autumn (September, October, November) - No. (%)	193 (25.4)	0 (0.0)	33 (23.9)	71 (24.0)	55 (31.8)	34 (22.7)
4) Winter (December, January, February) - No. (%)	189 (24.8)	1 (25.0)	28 (20.3)	84 (28.4)	31 (17.9)	45 (30.0)
Time of arrest						
1) Daytime (7:00–22:59) - No. (%)	596 (78.3)	3 (75.0)	110 (79.7)	228 (77.0)	139 (80.4)	116 (77.3)
2) Nighttime (23:00–6:59) - No. (%)	165 (21.7)	1 (25.0)	28 (20.3)	68 (23.0)	34 (19.7)	34 (22.7)
Region of arrest						
1) North - No. (%)	98 (12.9)	1 (25.0)	19 (13.8)	35 (11.8)	21 (12.1)	22 (14.7)
2) East - No. (%)	449 (59.0)	2 (50.0)	80 (58.0)	181 (61.2)	104 (60.1)	82 (54.7)
3) West - No. (%)	202 (26.5)	0 (0.0)	37 (26.8)	78 (26.4)	43 (24.9)	44 (29.3)
4) South - No. (%)	12 (1.6)	1 (25.0)	2 (1.5)	2 (0.7)	5 (2.9)	2 (1.3)
Type of advanced airway						
1) LMA - No. (%)	51 (6.7)	0 (0.0)	15 (10.9)	18 (6.1)	10 (5.8)	8 (5.3)
2) LT - No. (%)	603 (79.2)	4 (100.0)	114 (82.6)	250 (84.5)	134 (77.5)	101 (67.3)
3) ETT - No. (%)	107 (14.1)	0 (0.0)	9 (6.5)	28 (9.5)	29 (16.8)	41 (27.3)
EMS activity times						
Time from call to contact with patient, min						
- Mean (SD)	10.0 (5.8)	5.3 (1.0)	7.2 (1.7)	8.3 (2.2)	9.9 (2.8)	16.3 (9.6)
Time from call to AAM, min						
- Mean (SD)	18.9 (7.9)	5.3 (1.0)	10.5 (1.5)	15.4 (1.7)	21.0 (1.7)	31.6 (6.9)
Time from call to hospital arrival, min						
- Mean (SD)	37.5 (14.9)	24.3 (5.7)	30.5 (11.2)	33.6 (13.0)	38.9 (13.1)	50.4 (15.4)
Time from contact with patient to AAM, min						
- Mean (SD)	8.9 (5.5)	0.0 (0.0)	3.3 (2.1)	7.1 (2.4)	11.1 (3.2)	15.2 (6.8)
Time from contact with patient to hospital arrival, min						
- Mean (SD)	27.5 (12.9)	19.0 (5.8)	23.3 (10.7)	25.4 (12.6)	29.0 (12.7)	34.0 (13.1)
Time from AAM to hospital arrival, min						
- Mean (SD)	18.6 (12.5)	19.0 (5.8)	20.0 (11.0)	18.3 (12.9)	17.9 (13.0)	18.8 (12.5)

The data are expressed as the number (%) of patients, or the mean (SD), unless otherwise indicated.

Abbreviations, AAM, Advanced airway management, CPR, Cardiopulmonary resuscitation, ETT, Endotracheal tube, LMA, Laryngeal mask airway, LT, Laryngeal tube, PEA, pulseless electrical activity, SD, Standard deviation, VF, Ventricular fibrillation, VT, Ventricular tachycardia.

**Fig. 2:**
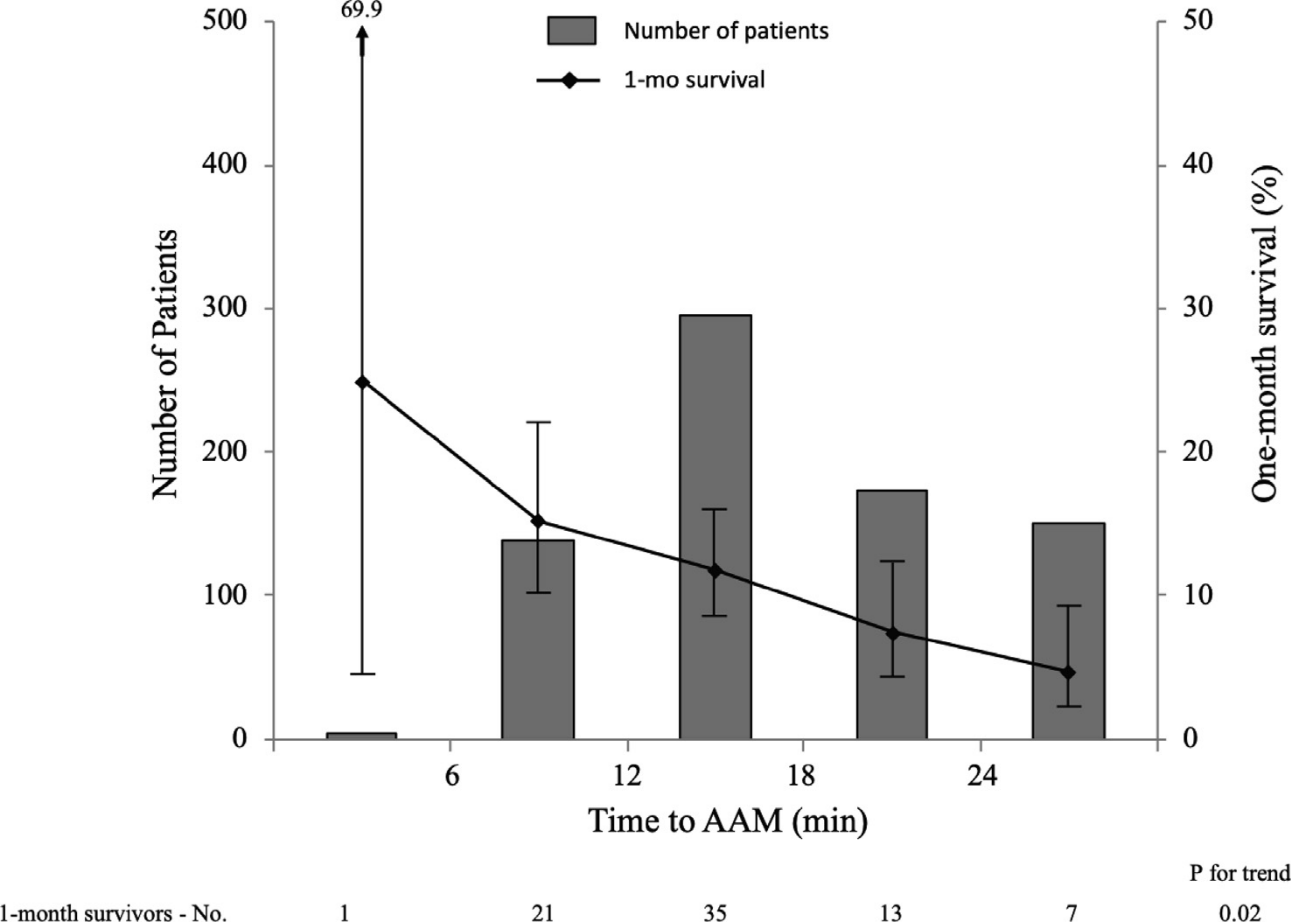
**Number of patients and unadjusted one-month survival according to 6-minute intervals of time to advanced airway management.** The mode of time to AAM was 13–18 min (N = 296). Delayed AAM was significantly associated with a decreased chance of one-month overall survival (Cochrane–Armitage trend test: P for trend = 0.02). The error bars represent 95% confidence intervals calculated using the Wilson score method. Abbreviations: AAM, Advanced airway management.

Overall, 77 (10.1%) survived one month after OHCA. Neurological outcomes were not available in 8 patients. Among the remaining 753 patients, 17 (2.3%) achieved favorable neurological outcomes one month after OHCA. Fig. 2 presents the number of patients and unadjusted one-month survival using 6-min intervals of time to AAM. Delayed AAM was significantly associated with a decreased chance of one-month survival (P for trend = 0.02). (The association between each additional minute of time to AAM and one-month survival is presented in Supplemental Figure.).

**Fig. 3:**
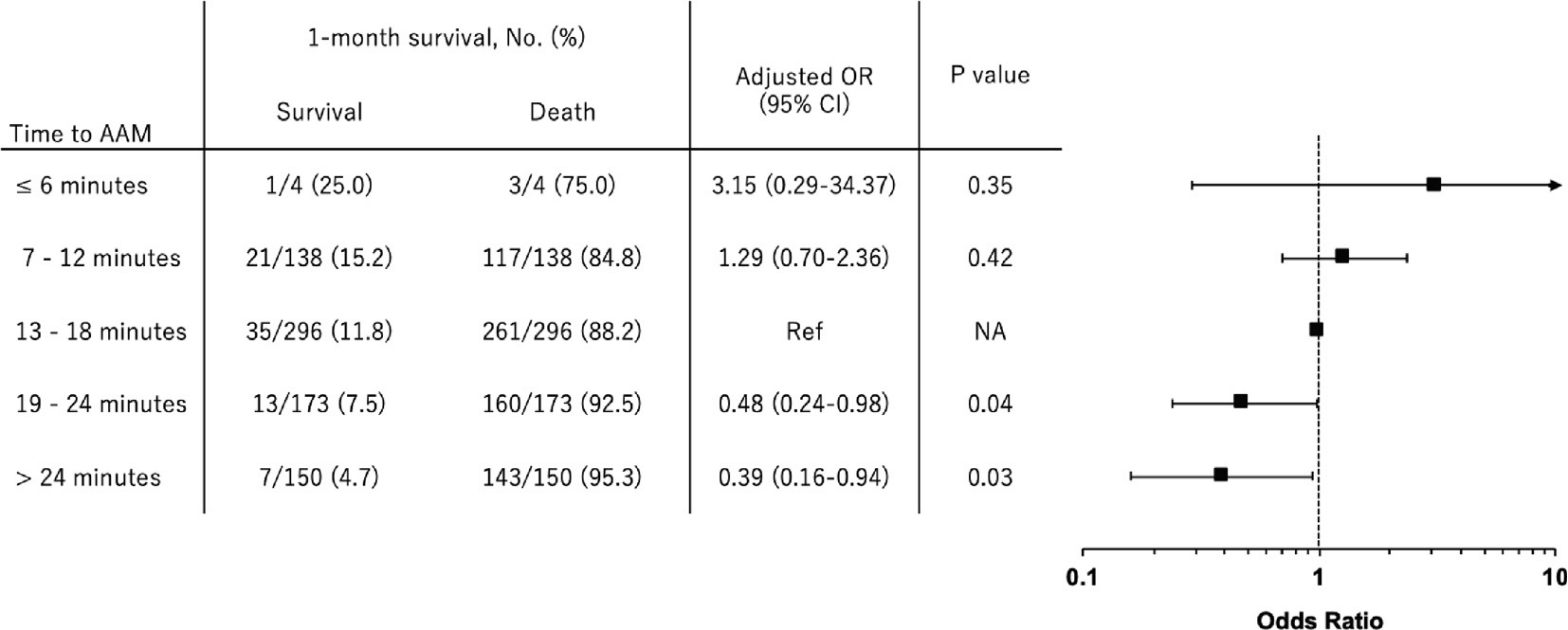
**Adjusted one-month survival according to 6-minute intervals of time to advanced airway management.** Compared with time to AAM from 13 to 18 min (the reference group), shorter time to AAM tended to be associated with a higher chance of survival (although not statistically significant), and longer time to AAM was significantly associated with a lower chance of survival. Abbreviations: AAM, Advanced airway management; CI, Confidence interval; OR, Odds ratio.

After multivariable adjustment, as presented in Fig. 3, compared with time to AAM from 13 to 18 min (the reference group), longer time to AAM was significantly associated with a lower chance of survival, whereas there were no statistically significant associations of shorter time to AAM with survival, although the point estimate favored shorter time to AAM. Table 2 shows the association between each additional minute of time to AAM and each outcome. Delay in AAM was associated with a decreased chance of one-month overall survival (multivariable adjusted OR per minute delay, 0.93 [95% CI, 0.89–0.97]; P = 0.001). Similar associations were observed for prehospital ROSC and neurologically favorable survival. Table 3 shows the results of the sensitivity and subgroup analyses. In the sensitivity analyses, all the models adjusted for different sets of variables showed similar results. In subgroup analyses, the association between time to AAM and one-month survival did not change regardless of age. In the ancillary analysis, in which time to AAM was divided into response time and procedure time, delayed response time was not associated with decreased survival (adjusted OR, 0.93 [95% CI, 0.87–1.00], P = 0.05), although delayed procedure time was significantly associated with decreased survival (adjusted OR, 0.93 [95% CI, 0.88–0.98], P = 0.008).

**Table 2:** Odds ratios of each outcome according to time to advanced airway management treated as a linear and continuous variable.

Outcome	No. of Patients with Favorable Outcome/Total Patients (%)	Crude OR (95%CI), *P* Value		Adjusted OR (95%CI), *P* Value	
**Primary Outcome**					
One-month survival	77/761 (10.1)	0.94 (0.90–0.97)	<0.001	0.93 (0.89–0.97)	0.001
**Secondary Outcomes**					
Prehospital ROSC	68/761 (9.0)	0.95 (0.91–0.98)	0.006	0.94 (0.90–0.99)	0.01
Neurologically favorable survival	17/753 (2.3)	0.89 (0.81–0.98)	0.02	0.83 (0.72–0.95)	0.006

In the multivariable logistic regression models, ORs were adjusted for gender, witness, bystander CPR, public-access defibrillation, dispatcher's instruction for CPR, initial rhythm, etiology of arrest, and type of advanced airway device.

Abbreviations: AAM, Advanced airway management; CI, Confidence interval; CPR, Cardiopulmonary resuscitation; OR, Odds ratio; ROSC, Return of spontaneous circulation.

**Table 3:** Sensitivity and Subgroup analyses.

	Adjusted OR per minute delay (95%CI), *P* Value	
**Sensitivity analysis (For time to AAM)**		
Model 1 (Adjusted for selected variables based on background knowledge)	0.93 (0.89–0.97)	0.001
Model 2 (Adjusted for selected variables based on univariable screening)	0.94 (0.90–0.98)	0.003
Model 3 (Adjusted for all available variables that could influence survival)	0.93 (0.89–0.97)	0.002
**Subgroup analysis (For time to AAM)**		
<8 years	0.89 (0.79–0.99)	0.04
≥8 years	0.94 (0.89–0.98)	0.01
**Ancillary analysis (Time to AAM was divided into the following 2 intervals)**		
Response time (from call to contact with patient)	0.93 (0.87–1.00)	0.05
Procedure time (from contact with patient to AAM)	0.93 (0.88–0.98)	0.008

In Model 1, the ORs were adjusted for gender, witness, bystander CPR, public-access defibrillation, dispatcher's instruction for CPR, initial rhythm, etiology of arrest, and type of advanced airway device.

In Model 2, the ORs were adjusted for age, witness, bystander CPR, public-access defibrillation, initial rhythm, etiology of arrest, time of arrest, and physician involvement in prehospital ALS.

In Model 3, the ORs were adjusted for age, gender, witness, bystander CPR, public-access defibrillation, dispatcher's instruction for CPR, initial rhythm, etiology of arrest, year of arrest, season of arrest, time of arrest, region of arrest, physician involvement in prehospital ALS, and type of advanced airway device.

In ancillary analysis, time to AAM was divided into response time and procedure time.

Abbreviations: AAM, Advanced airway management; ALS, Advanced life support; CI, Confidence interval; CPR, Cardiopulmonary resuscitation; OR, Odds ratio.

## Discussion

Our study found that delayed AAM was associated with a decreased chance of one-month overall survival among pediatric patients with OHCA who received AAM by EMS personnel in the prehospital setting. This association was consistently demonstrable with multiple different statistical analyses. Although the observational study design precludes ascertainment of causality, the statistical robustness of our findings that early AAM may be beneficial in OHCA is ensured by the consistency demonstrated by multiple statistical analyses, as well as the use of government-led nationwide population-based registry data that routinely collected for all pediatric OHCA patients who were transported to an emergency hospital.

No RCTs or observational studies have directly examined the optimal timing of AAM during pediatric cardiac arrest. As for adult cardiac arrest, although there have been no RCTs, several observational studies have been conducted on this topic.29., 31., 32. The largest observational study, which we conducted previously and included approximately 165,000 adult OHCA patients, demonstrated that delayed AAM was associated with a decreased chance of one-month survival (adjusted OR per minute delay, 0.90 [95% CI, 0.90–0.91]).^19^ The findings of this pediatric study are consistent with those of previous studies.

In this study, delay in AAM, especially over 19 minutes from emergency call, was significantly associated with poor outcomes (Fig. 3). Our findings raise the interesting question of whether early AAM is effective in pediatric OHCA compared with no AAM or non-early (including no and delayed) AAM, although this study was not designed to evaluate whether an advanced airway should be placed for pediatric OHCA. Prehospital AAM may have an important role in children with OHCA because the majority of pediatric OHCA cases have a respiratory (asphyxial) etiology.8., 9., 10., 11. In fact, our study demonstrated that patients with non-cardiac etiology accounted for as many as 76.7% of the pediatric OHCA cohort (Table 1). However, previous studies have never shown the superiority of AAM in pediatric resuscitation, nor did a previous large RCT on adult OHCA.^15^ The reason why the adult RCT failed to detect a difference in survival between ETI and BVM ventilation may be due to the lack of consideration regarding the timing of AAM (i.e., the median time to initiation of ALS was 20 min in this RCT), despite the potential for greater harm with delayed treatment. Further study is required to determine whether AAM is effective for pediatric OHCA in the context of the timing of AAM.

Our findings have important implications for EMS systems and prehospital care for OHCA. Although earlier treatment may encompass many variables (e.g., high-quality CPR or improved team performance) other than time itself, approaches to shortening time to AAM (e.g., organizing EMS systems to achieve early AAM, omitting instruction of medical directors, improving skill in advanced airway placement, or increasing physician-staffed ambulances) have the potential to improve outcomes after OHCA. As time to AAM could be divided into response time and procedure time, we further performed analyses at each time point. Although delays in both response time and procedure time tended to be associated with a decreased chance of one-month survival, there was no statistically significant difference in response time (Table 2). In pediatric resuscitation, procedure time may have a greater impact on survival than response time, although we acknowledge that the OR with 95% CIs for response time may have been statistically different with a larger sample size.

## Limitations

Several limitations of our study should be considered when interpreting the results of our study. First, despite efforts to control for selection bias and confounders using a variety of analytical techniques, observational study design could not necessarily derive causality because of unmeasured confounding and residual selection bias. Additionally, resuscitation time bias may have occurred in this study. In an observational study on cardiac arrest, the longer the resuscitation time, the more likely the occurrence of exposure. This phenomenon tends to bias the results toward a detrimental effect, as the duration of CPR is strongly associated with poor outcomes.^33^

Second, the generalizability of our findings may be limited. As our analysis did not include neonates and infants, it is unclear whether the findings are applicable to them. We excluded such population from our analysis due to potential differences in physiological characteristics and etiology of cardiac arrest, as in previous studies.3., 4. The generalizability of our findings to patients under 8 years may also be limited. In our subgroup analysis, delayed AAM was associated with decreased survival, regardless of age (<8 or ≥8 years). However, as several municipalities restrict AAM to the patients aged >8 years, the results derived from the subgroup of patients aged under 8 years may be biased. It is also unclear whether the findings of our study are applicable to other countries. Different airway management protocols or training systems in EMS may produce different results. In Japan, SGA, especially the esophageal obturator airway, is the most frequently chosen as the AAM device. This choice may differ from that of other countries.34., 35.

Third, data on failed AAM attempts were not available in this registry because only successful attempts were recorded in the database. Although failed attempts are likely to be associated with poor outcomes, the exclusion of patients with ultimately failed AAM attempts could potentially distort our results.

Fourth, misclassification of the time variables could have occurred as time variables were classified in whole minutes, although it is likely that such a potential misclassification is undifferentiated.

Fifth, despite the nationwide population-based study design including multi-year events, the sample size and number of patients with favorable outcomes were not very large. When further dividing the included patients into several categories, the number of patients in each category could decrease, reducing the likelihood of detecting any statistically significant differences. Thus, we could not investigate neurological outcomes, except for the case when time to AAM was treated as a linear and continuous variable, or we could not conduct a subgroup analysis based on the type of airway device. In addition, the 95% CIs for the point estimates for the treatment effect overlapped unity in some analyses but may have been statistically different with a larger sample size. An adequately powered RCT is required to clearly determine the optimal timing of AAM for pediatric OHCA.

Finally, the possibility remains that time to AAM is merely a surrogate for other resuscitation efforts (e.g., improved team performance), accessibility to ALS (e.g., epinephrine), difficulty in resuscitation because of comorbidities (e.g., obesity), or resuscitation time itself. Further studies prospectively collecting such detailed data are required to determine more precisely whether delayed AAM is associated with poor outcomes after OHCA.

## Conclusion

In this nationwide population-based study from 2014 to 2019 in Japan, delayed AAM was associated with a decreased chance of one-month survival among children with OHCA who received prehospital AAM, although the influence of resuscitation time bias might remain.

## Conflict of Interest

None.

## Figures and Tables

**Supplementary Fig. 1:**
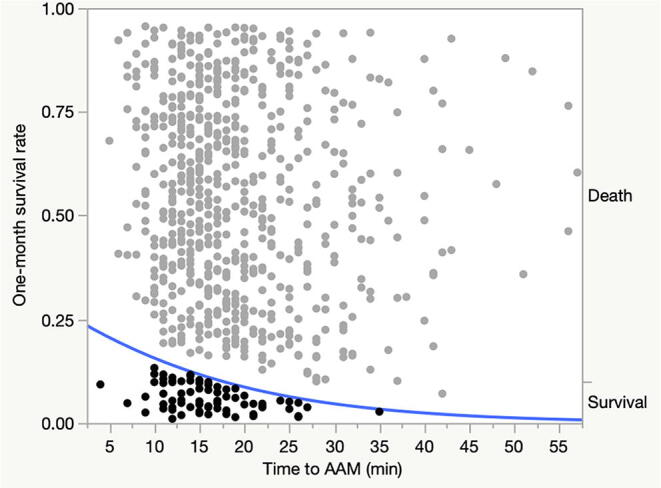
**The association between each additional minute of time to AAM and one-month surviva.** Abbreviations: AAM, Advanced airway management.
